# CD276 and the gene signature composed of GATA3 and LGALS3 enable prognosis prediction of glioblastoma multiforme

**DOI:** 10.1371/journal.pone.0216825

**Published:** 2019-05-10

**Authors:** Yasuo Takashima, Atsushi Kawaguchi, Azusa Hayano, Ryuya Yamanaka

**Affiliations:** 1 Laboratory of Molecular Target Therapy for Cancer, Graduate School for Medical Science, Kyoto Prefectural University of Medicine, Kyoto, Japan; 2 Center for Comprehensive Community Medicine, Faculty of Medicine, Saga University, Saga, Japan; University of South Alabama Mitchell Cancer Institute, UNITED STATES

## Abstract

Glioma is the most common type of primary brain tumor, accounting for 40% of malignant brain tumors. Although a single gene may not be a marker, an expression profiling and multivariate analyses for cancer immunotherapy must estimate survival of patients. In this study, we conducted expression profiling of immunotherapy-related genes, including those in Th1/2 helper T and regulatory T cells, and stimulatory and inhibitory checkpoint molecules associated with survival prediction in 571 patients with malignant and aggressive form of gliomas, glioblastoma multiforme (GBM). Expression profiling and Random forests analysis of 21 immunosuppressive genes and Kaplan-Meier analysis in 158 patients in the training data set suggested that CD276, also known as B7-H3, could be a single gene marker candidate. Furthermore, prognosis prediction formulas, composed of Th2 cell-related GATA transcription factor 3 (GATA3) and immunosuppressive galactose-specific lectin 3 (LGALS3), based on 67 immunotherapy-related genes showed poor survival with high scores in training data set, which was also validated in another 413 patients in the test data set. The CD276 expression helped distinguish survival curves in the test data set. In addition, inhibitory checkpoint genes, including T cell immunoreceptor with Ig and ITIM domains, V-set domain containing T cell activation inhibitor 1, T-cell immunoglobulin and mucin-domain containing 3, and tumor necrosis factor receptor superfamily 14, showed potential as secondary marker candidates. These results suggest that CD276 expression and the gene signature composed of GATA3 and LGALS3 are effective for prognosis in GBM and will help us understanding target pathways for immunotherapy in GBM.

## Introduction

Glioma is the most common type of primary brain tumor accounting for 40% of all malignant brain tumors [1]. The World Health Organization (WHO) classifies gliomas into grades I-IV by malignancy and overall survival (OS) [1]. Glioblastoma multiforme (GBM) is a fast-growing grade IV malignant glioma [1]. GBM is the most common brain tumor in adults with a median OS of only 9–15 months and a 5-year survival rate of only 9.8%, in spite of treatment with radiotherapy, chemotherapy, and surgery [2–4]. Therefore, early diagnosis and prognosis are required for the accurate treatment of GBM.

Recent trends of studies on central nervous system tumors have focused on cancer immunotherapy, which target immune checkpoint molecules on the surfaces of tumors, antigen-presenting cells (APCs), and T cells [5]. The monoclonal antibodies bind to programmed cell death protein-1 (PD-1), cytotoxic T-lymphocyte-associated antigen-4 (CTLA4), and indoleamine-2,3-dioxygenase 1 (IDO1), thereby, relieving T-cells from immunosuppression by tumor cells [6,7]. The PD-1 receptor on T cells interacts with PD-1 ligands including PD-L1 (CD274) and PD-L2 (PDCD1LG2), on the surface of tumor cells [8]. In the context of tumor, major histocompatibility complex (MHC) class-I antigen presenting cells, a ligand interaction with PD-1 induces suppression of T cells by inhibiting T-cell tumor lysis [8]. Similarly, CTLA4 is also a highly potent inhibitory T-cell receptor and preferentially interacts with both CD80 (B7-H1) and CD86 (B7-H2) receptors on APCs. This prevents the CD28 stimulatory receptor from binding on T cells and increases interleukin (IL)-6 production [9–11]. IDO-expressing macrophages and dendritic cells regulate T-cell metabolism and response via kynurenine signaling in tryptophan oxidation [12], aryl hydrocarbon receptor (AhR), mammalian target of rapamycin (mTOR) signaling, and general control nonderepressible 2 (GCN2) in serine/threonine kinetics [13].

The study on glioma reported PD-L1 expression in the patients’ characteristics and the data were deposited in both Chinese Glioma Genome Atlas (CGGA) and The Cancer Genome Atlas (TCGA), however, it was hard to calculate the prognoses of the patients in mixed stages II-IV, primary, recurrence, and progressive gliomas [14]. On the other hand, only in primary GBM, grade IV glioma, the study on the balance of T helper type 1 (Th1) and Th2 cells associated with the PD-1 axis succeeded in estimating their prognoses. Thus, it is important to examine T-cell status coupled with the PD-1 axis in prognosis of GBM [15]. Here we examined the expression of 67 immunotherapy-related genes involved in T-cell status and checkpoint molecules, especially 21 inhibitory checkpoint molecules, in 571 non-treated primary GBM patients. The results proposed a single gene marker candidate CD276 (B7-H3) and the gene signature composed of GATA3 transcription factor and galectin-3 (LGALS3) in multivariate analyses. The genes identified in the study could serve as novel candidate targets for immunotherapies and would help us understand target pathways in GBM.

## Methods

### Data set

RNA-Seq data and clinical information in glioblastoma multiforme (GBM) obtained from The Cancer Genome Atlas (TCGA) (NIH, https://cancergenome.nih.gov/) were used. Training data set and test data set comprised 158 samples derived from Glioblastoma Multiforme (TCGA, Provisional) (https://www.cbioportal.org/study?id=gbm_tcga) and 413 samples derived from Molecular Profiling Reveals Biologically Discrete Subsets and Pathways of Progression in Diffuse Glioma, 2016 (https://tcga-data.nci.nih.gov/docs/publications/lgggbm_2015/) available for survival analysis and expression profiling, respectively [15].

### Kaplan-Meier analysis

The Kaplan-Meier analysis was performed to estimate survival distributions for subgroups with log-rank test among subgroups using JMP (SAS Institute Inc., Tokyo, Japan) [16].

### Random survival forests analysis

Random survival forests analysis was performed to determine variable importance factors distinguishing expression of genes with survival times, which was estimated by randomly permutating its values and recalculating the predictive accuracy of the model that were expressed as the log rank test statistics using the randomForestSRC package in R [17].

### Cox hazards regression analysis

Correlations between gene expression and survival times were evaluated by multivariate analyses. Clinical characteristics were used as an additional variable to execute multivariate analyses. The statistical data was determined by the Cox proportional hazards regression model using JMP (SAS Institute Inc.) [18].

### Multivariate correlation coefficient analysis

Correlation among variables were analyzed using the glasso package in R [19,20].

### Statistics

Statistical analysis was performed using R software, Bioconductor, and JMP (SAS Institute Inc.) [15]. P < 0.05 was considered statistically significant.

## Results

### Patient characteristics

The aim of this study was the discovery of novel prognosis markers and gene signatures from a viewpoint of cancer immunotherapy, especially inhibitory checkpoints, based on the expression data and clinical information in two independent GBM data set. Therefore, we arranged for the gene set related to cancer immunotherapy including type 1 T helper cells, type 2 T helper cells, regulatory T cells, stimulatory checkpoint, and inhibitory checkpoint (S1 Table). This study analyzed data for 571 non-treated primary GBM patients (WHO grade IV), deposited in TCGA (Table 1, S1 Fig). The training data set constituted of 158 patients from the Glioblastoma Multiforme data set, as shown in S1A Fig. The median OS was 11.25 months (range: 0.16–88.07) (S1C Fig). The median age was 60 years (range: 21–89). Age ≥ 60 (99 patients, 62.6%) showed poor prognosis than age < 60 (59 patients, 37.3%), with hazard ratio (HR) by 1.36 (P < 0.1107). The number of male and female patients was 102 (64.5%) and 56 (35.4%) respectively; they showed no difference in OS (HR = 1.03, P = 0.4699). The median score of the preoperative Karnofsky performance status (KPS) was 80 (range: 40–100); KPS ≥ 70 (89 patients, 74.7%) and the KPS < 70 (30 patients, 25.2%) showed no difference in OS (HR = 0.93, P = 0.7984). Patients were monitored for tumor recurrence during the initial and maintenance therapies by using magnetic resonance imaging or computed tomography. There were 13 patients who had cancer recurrence. The test data set constituted of 413 patients from the Merged Cohort of LGG and GBM data set, as shown in S1B Fig. The median OS was 11.3 months (range: 0.1–127.5) (S1D Fig). The median age was 58 years (range: 10–88). Age ≥ 60 (238 patients, 57.6%) showed poor prognosis than age < 60 (175 patients, 42.3%) with HR by 2.18 (P < 0.0001). The numbers of male and female patients were 243 (58.9%) and 170 (41.0%), respectively; they showed no difference in OS (HR = 1.2, P = 0.1095). The median score of KPS was 80 (range: 20–100); KPS ≥ 70 (237 patients, 77.1%) showed better prognosis than KPS < 70 (70 patients, 22.8%) with HR by 0.45 (P < 0.0001).

**Table 1:** Patient characteristics of glioblastoma multiform.

**Training data set (N = 158)**							
	**N (%)**	**Median (Min—Max)**	**Multivariate analysis for OS**^a^				
				**Median (Min—Max)**	**HR**^b^	**95% CI**^c^	**P-value**
**Total**	**158 (100)**		**OS (days)**:	**337 (5–2642)**			
**Age (years)**	**158 (100)**	**60 (21–89)**					
**Age > 60**	**99 (62.6)**		**OS (days)**:	**272 (6–2095)**	**1.36**	**0.93–2.02**	**0.1107**
**Age < 60**	**59 (37.3)**		**OS (days)**:	**408 (5–2642)**	**1**		
**Gender**	**158 (100)**						
**Male**	**102 (64.5)**		**OS (days)**:	**314.5 (5–2642)**	**1.03**	**0.73–1.54**	**0.7699**
**Female**	**56 (35.4)**		**OS (days)**:	**273.5 (6–1458)**	**1**		
**KPS**^d^	**119 (100)**	**80 (40–100)**					
**KPS > 70**	**89 (74.7)**		**OS (days)**:	**331 (13–1458)**	**0.93**	**0.53–1.70**	**0.7984**
**KPS < 70**	**30 (25.2)**		**OS (days)**:	**192 (26–1448)**	**1**		
**Test data set (N = 413)**							
	**N (%)**	**Median (Min—Max)**	**Multivariate analysis for OS**				
				**Median (Min—Max)**	**HR**	**95% CI**	**P-value**
**Total**	**413 (100)**		**OS (days)**:	**339 (3–3825)**			
**Age (years)**	**413 (100)**	**58 (10–88)**					
**Age > 60**	**238 (57.6)**		**OS (days)**:	**250.5 (3–3615)**	**2.18**	**1.74–2.73**	**<0.0001**
**Age < 60**	**175 (42.3)**		**OS (days)**:	**477 (12–3825)**	**1**		
**Gender**	**413 (100)**						
**Male**	**243 (58.9)**		**OS (days)**:	**363 (3–3474)**	**1.2**	**0.96–1.50**	**0.1095**
**Female**	**170 (41.0)**		**OS (days)**:	**288 (3–3825)**	**1**		
**KPS**	**307 (100)**	**80 (20–100)**					
**KPS > 70**	**237 (77.1)**		**OS (days)**:	**432 (3–3825)**	**0.45**	**0.33–0.62**	**<0.0001**
**KPS < 70**	**70 (22.8)**		**OS (days)**:	**207 (6–1791)**	**1**		

NOTE:

^a^OS; overall survival,

^b^HR; hazard ratio,

^c^CI; confidence interval,

^d^KPS; Karnofsky performance status.

### Multivariate analyses for the genes encoding inhibitory checkpoint molecules

**Fig 1:**
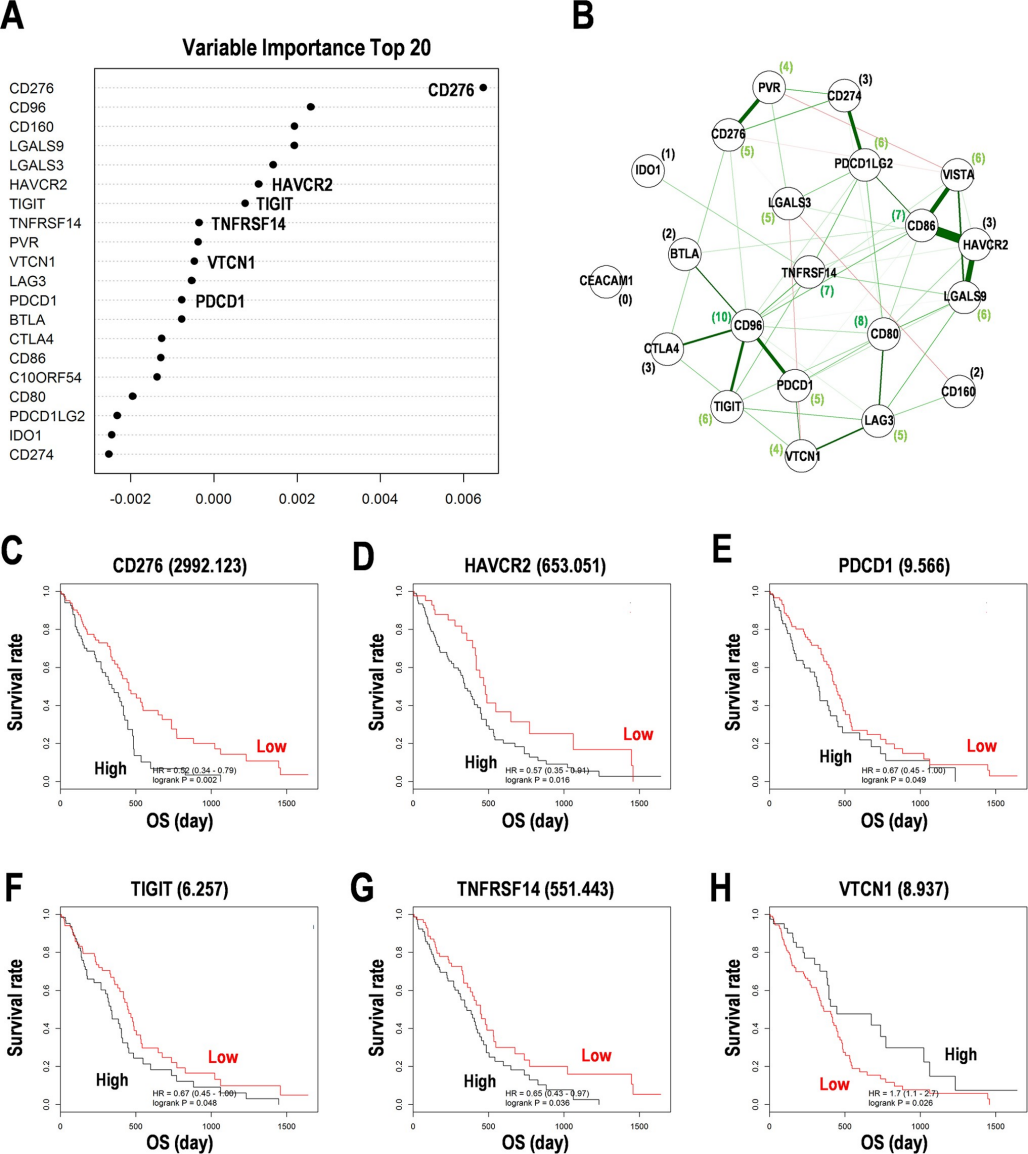
Prognostic marker candidates for immunosuppression in the training data set of glioblastoma multiform (GBM). (**A**) Random survival forests analysis for immunosuppressive pathway genes in GBM. Top 20 of variable importance were shown. (**B**) Graphical lasso estimation for the genetic interaction of immunosuppressive pathway genes in GBM. Schematics were drawn by glasso package in R. Numbers in the parentheses indicate the numbers of genetic interaction. Thick and thin lines indicate strong and weak interaction, respectively. (**C-H**) Kaplan-Meier survival analysis for immunosuppressive pathway genes in GBM. (**C**) CD276/B7-H3. (**D**) HAVCR2/TIM-3. (**E**) PDCD1/PD-1. (**F**) TIGIT. (**G**) TNFRSF14. (**H**) VTCN1. Numbers in the parentheses indicate the threshold of gene expression. High and low indicate subgroups with over and under the threshold. OS, overall survival. HR, hazard ratio. Subgroups were divided by the median expression of genes.

First, we examined whether the genes encoding inhibitory checkpoint molecules could serve as prognosis markers (S1 Table). The expression values of 21 immunosuppressive genes were obtained from 158 GBM patients, which were derived from post-RNA-Seq of the resected tumors and biopsies. Since the expression values were relatively compact, analyses were done using the values of fragments per kilobase of exon per million reads mapped (FPKM). Random survival forests analysis returned variable importance of each gene in the training data set (Fig 1A). Especially, CD276 (B7-H3), CD96, CD160, LGALS3, LGALS9, HAVCR2 (TIM-3), and TIGIT were associated with relatively high scores, followed by TNFRSF14, PVR, VTCN1, LAG3, PDCD1 (PD-1), and BTLA (Fig 1A), while CTLA-4, CD86, CD80, CD274 (PD-L1), VISTA (C10orf54), PDCD1LG2 (PD-L2), and IDO1 were associated with relatively low scores (Fig 1A). Graphical lasso analysis was performed to investigate genetic interaction among the 21 immunosuppressive genes (Fig 1B). The results demonstrated that CD96, CD80, CD86, and TNFRSF14 constitute a network hub (node ≥ 7), interacting with PDCD1LG2, VISTA, LGALS9, TIGIT, LGALS3, CD276, LAG3, PDCD1, PVR, and VTCN1 (node ≤ 6) (Fig 1B). Moreover, we tried to develop a prognosis prediction formula using the 21 immunosuppressive genes, whereas a Cox hazard regression analysis returned only CD276 (S2 Fig). Higher expression of CD276 showed poor prognosis (HR = 1.923, P = 0.002) (Fig 1C). Besides, higher expression of HAVCR2, PDCD1, TIGIT, and TNFRSF14, and lower expression of VTCN1 also showed poor prognosis (HR > 1.495, P < 0.05) (Fig 1D–1H). These results suggest that CD276 and TIGIT could contribute to prognosis in several analyses and construction of immunosuppressive genetic networks in the training data set of GBM.

### Multivariate analyses for the genes involved in cancer immunotherapy in the training data set

**Fig 2:**
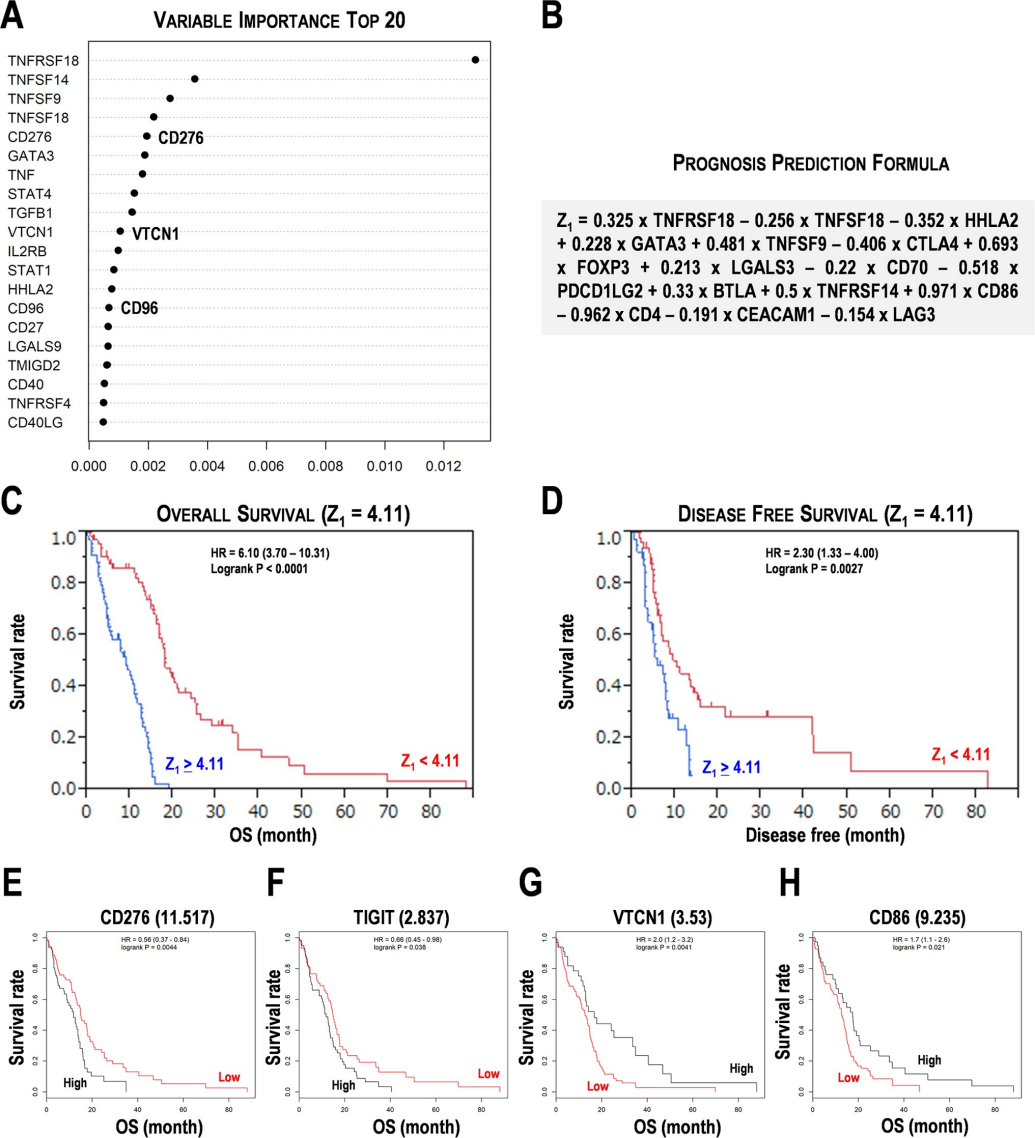
Prognostic marker candidates for cancer immunotherapy in the training data set of glioblastoma multiform (GBM). (**A**) Random survival forest analysis for cancer immunotherapy-related genes in GBM. Top 20 of variable importance was shown. (**B**) Prognosis prediction formula for cancer immunotherapy-related genes in GBM. The Z_1_ score is calculated by the expression values of 16 immunosuppressive pathway genes. (**C,D**) Kaplan-Meier survival analysis using the Z_1_ score = 4.11. (**C**) Overall survival analysis (OS). (**D**) Disease free survival analysis. HR, hazard ratio. (**E-H**) Kaplan-Meier survival analysis for representative immunosuppressive pathway genes in GBM. (**E**) CD276/B7-H3. (**F**) TIGIT. (**G**) VTCN1. (**H**) CD86. Numbers in the parentheses indicate the threshold of gene expression. High and low indicate subgroups with over and under the threshold. OS, overall survival. HR, hazard ratio. Subgroups were divided by the median score of Z_1_ and the median expression of genes.

Second, we analyzed 67 immunotherapy pathway-related genes that include 17 Th1-related genes, 18 Th2-related genes, 21 stimulatory checkpoint genes, 21 inhibitory checkpoint genes, and 14 regulatory T cells (Treg)-related genes, thereby, focusing on the immunosuppressive genes to identify prognosis markers (Fig 2, S1 Table). Since the expression values were expanded in the genes variously categorized, the values were converted into a log scale by log_2_(x+1). Random survival forests analysis returned variable importance of each gene in the training data set (Fig 2A). Especially, TNFRSF18, TNFSF14, TNFSF9, TNFSF18, CD276, GATA3, TNF, STAT4, and TGFB1 were associated with relatively high scores (Fig 2A). Immunosuppressive genes including CD276, VTCN1, and CD96 were ranked by relatively high score of the variable importance (Fig 2A). Cox proportional hazards regression analysis resulted in 16 candidate genes being associated with the effect of variables on the OS (S3 Fig). Based on the results, a prognosis prediction formula was constructed as follows (Fig 2B): Z_1_ = 0.325 × TNFRSF18–0.256 × TNFSF18–0.352 × HHLA2 + 0.228 × GATA3 + 0.481 × TNFSF9–0.406 × CTLA4 + 0.693 × FOXP3 + 0.213 × LGALS3–0.22 × CD70–0.518 × PDCD1LG2 + 0.33 × BTLA + 0.5 × TNFRSF14 + 0.971 × CD86–0.962 × CD4–0.191 × CEACAM1–0.154 × LAG3.

The 158 GBM patients were divided by the median score of Z_1_ by 4.11. The subgroup with Z_1_ ≥ 4.11 showed poor prognosis for OS (HR = 6.10, P < 0.0001) (Fig 2C) and disease-free survival (HR = 2.30, P = 0.0027) (Fig 2D). In immunosuppressive genes, higher expression of CD276 (HR = 1.785, P = 0.0044) and TIGIT (HR = 1.515, P = 0.038) showed poor prognosis (Fig 2E and 2F). On the contrary, lower expression of VTCN1 (HR = 2.0, P = 0.0041) and CD86 (HR = 1.7, P = 0.021) showed poor prognosis (Fig 2G and 2H). Besides, higher expression of CD163, FOXP3, GATA3, IL18R1, TGFB3, TGFB1, TNFRSF18, TNFSF14, and TNFSF4 also showed poor prognosis (HR > 1.492, P < 0.05) (S4A–S4I Fig). On the contrary, lower expression of HHLA2, STAT1, and TBX21 also showed poor prognosis (HR > 1.6, P < 0.05) (S4J–S4L Fig). These results suggested that the formula Z_1_ is effective in estimating prognosis in the training data set. Besides, Kaplan-Meier analyses suggested that expression of the genes involved in Th2 cells, Treg, and stimulatory checkpoint molecules and suppression of the genes involved in Th1 cells would result in poor prognosis.

### Multivariate analyses for the genes involved in cancer immunotherapy in the test data set

**Fig 3:**
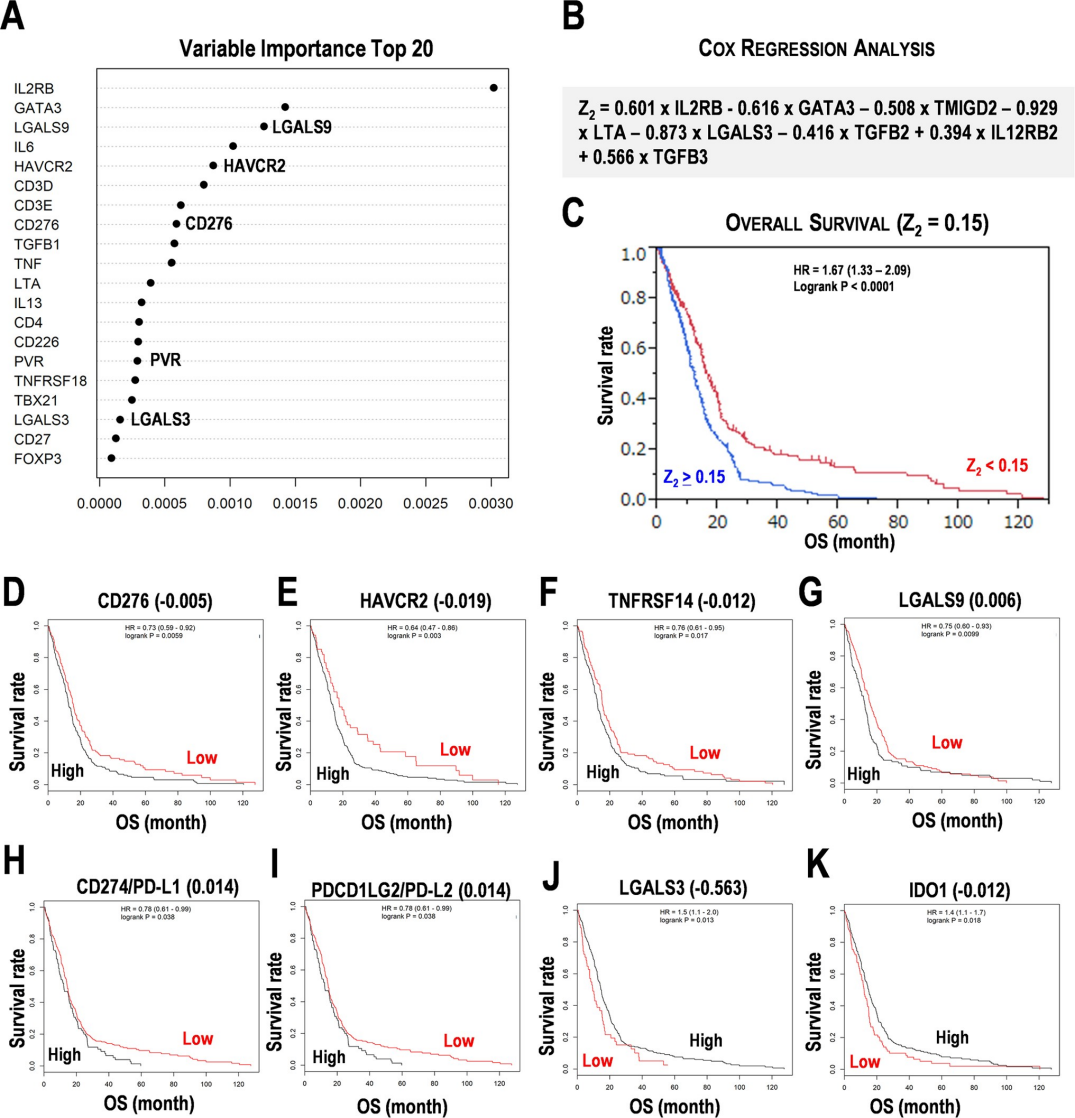
Prognostic marker candidates for cancer immunotherapy in the test data set of glioblastoma multiform (GBM). (**A**) Random survival forest analysis for cancer immunotherapy-related genes in GBM. Top 20 of variable importance was shown. (**B**) Prognosis prediction formula for cancer immunotherapy-related genes in GBM. The Z_2_ score is calculated by the expression values of 8 immunosuppressive pathway genes. (**C**) Kaplan-Meier survival analysis for overall survival (OS) using the Z_2_ score = 0.15. HR, hazard ratio. (**D-H**) Kaplan-Meier survival analysis for representative immunosuppressive pathway genes in GBM. (**D**) CD276/B7-H3 (**E**) HAVCR2/TIM-3. (**F**) TNFRSF14. (**G**) LGALS9. (**H**) CD274/PD-L1. (**I**) PDCD1LG2/PD-L2. (**J**) LGALS3. (**K**) IDO1. Numbers in the parentheses indicate the threshold of gene expression. High and low indicate subgroups with over and under the threshold. OS, overall survival. HR, hazard ratio. Subgroups were divided by the median score of Z_2_ and the median expression of genes.

Similarly, we also analyzed the immunotherapy pathway-related genes using log scale-expression values in the test data set (Fig 3). Random survival forests analysis returned variable importance of each gene (Fig 3A). Especially, IL12RB, GATA3, LGALS9, IL6, HAVCR2, CD3D, CD3E, CD276, TGFB1, and TNF were associated with relatively high scores (Fig 3A). Immunosuppressive genes including LGALS9, HAVCR2, CD276, PVR, and LGALS3 were ranked in the top 20 genes (Fig 3A). Cox hazards regression analysis returned 8 candidates genes associated with the effect of variables upon the OS (S5 Fig). Based on the results, a prognosis prediction formula was constructed as follows (Fig 3B): Z_2_ = 0.601 × IL2RB—0.616 × GATA3–0.508 × TMIGD2–0.929 × LTA—0.873 × LGALS3–0.416 × TGFB2 + 0.394 × IL12RB2 + 0.566 × TGFB3.

The 413 GBM patients were divided by the median score of Z_2_ = 0.15. The subgroup with Z_2_ ≥ 0.15 showed poor prognosis for OS (HR = 1.67, P < 0.0001) (Fig 3C). For immunosuppressive genes, higher expression of CD276 (HR = 1.369, P = 0.0059), HAVCR2 (HR = 1.562, P = 0.003), TNFRSF14 (HR = 1.315, P = 0.017), LGALS9 (HR = 1.333, P = 0.0099), CD274 (HR = 1.282, P = 0.038), and PDCD1LG2 (HR = 1.282, P = 0.038) showed poor prognosis (Fig 3D–3I). On the contrary, lower expression of LGALS3 (HR = 1.5, P = 0.013) and IDO1 (HR = 1.4, P = 0.018) showed poor prognosis (Fig 3J and 3K). Besides, higher expression of CSF2, IL12RB2, IL13, IL2RB, IL3, IL4, IL5, IL6, IL9, TBX21, TGFB1, TNFRSF18, and TNFRSF4 also showed poor prognosis (HR > 1.315, P < 0.05) (S6A–S6M Fig). On the contrary, lower expression of CD3D, CD3E, CD3G, GATA3, LTA, STAT1, STAT4, and TNF also showed poor prognosis (HR > 1.3, P < 0.05) (S6N–S6U Fig). These results suggest that the formula Z_2_ is effective for estimating prognosis in the test data set. Besides, Kaplan-Meier analyses suggested that expression of the genes involved in Th2 cells, stimulatory checkpoint molecules, and suppression of the genes involved in Th1 cells would result in poor prognosis.

### The gene signature for prognosis prediction in glioblastoma multiforme

**Fig 4:**
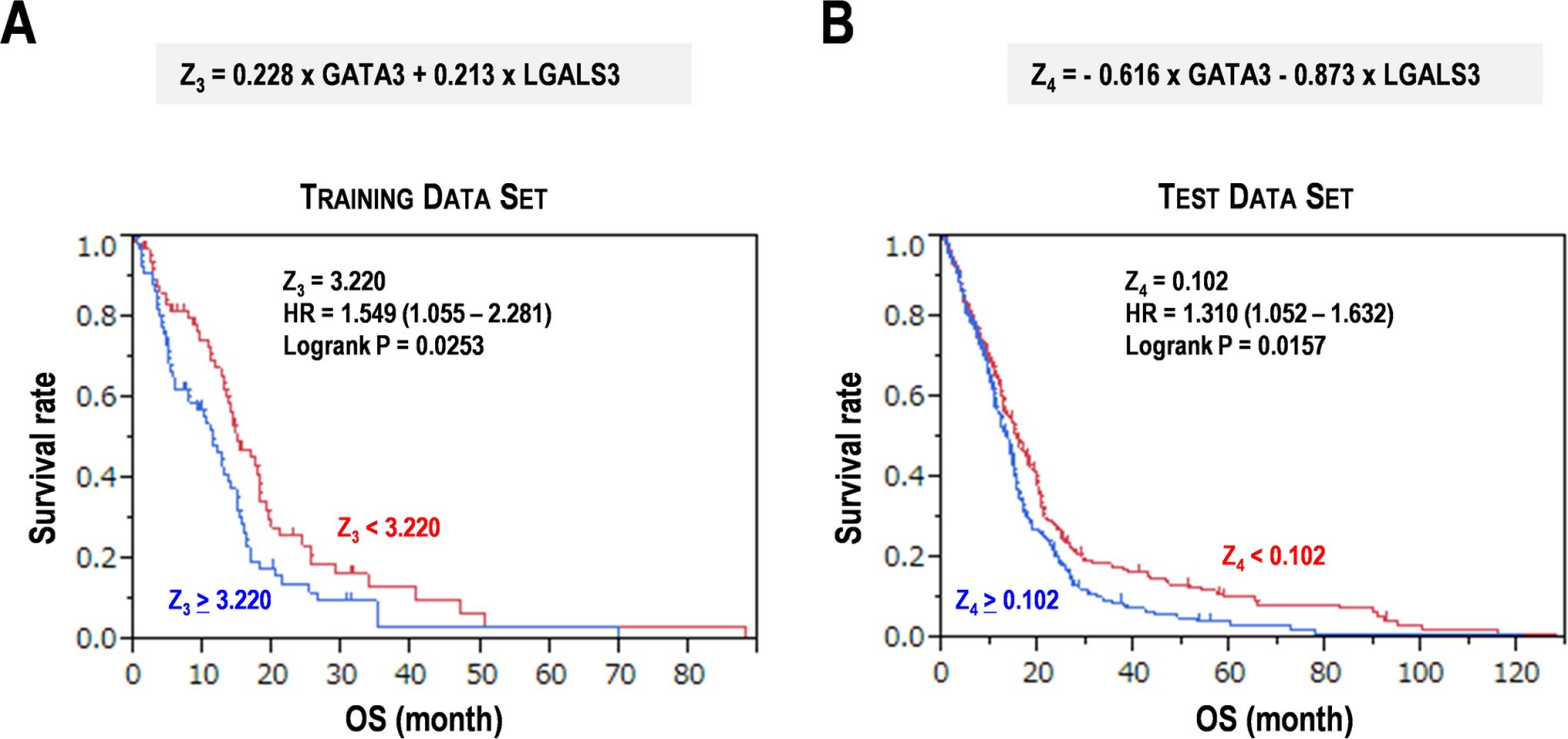
Gene signature constituted of Th2 cell-related gene GATA3 and immunosuppressive gene LGALS3 in glioblastoma multiform (GBM). (**A**) Survival distribution using the Z_3_ score in the training data set of GBM. (**B**) Survival distribution using the Z_4_ score in the test data set of GBM. OS, overall survival. HR, hazard ratio. Subgroups were divided by the median scores of Z_3_ and Z_4_.

We tested whether the prognosis prediction formula Z_1_ derived from the training data set and Z_2_ derived from test data set are effective to estimate prognosis in the test data set and training data set, respectively. However, the subgroup with Z_1_ ≥ -0.064 (median score) showed no difference in OS (HR = 0.853, P = 0.1554) in the test data set (S7A Fig). Similarly, the subgroup with Z_2_ ≥ -11.118 (median score) also showed no differences in OS (HR = 0.735, P = 0.12) in the test data set (S7B Fig). Then, we next focused on the common factors, including the GATA3 transcription factor and the LGALS3 β-galactoside-binding protein family, in the both data sets, thereby, constructed the formulas as follows (Fig 4A and 4B): Z_3_ = 0.228 × GATA3 + 0.213 × LGALS3 in the training data set, and Z_4_ = − 0.616 × GATA3–0.873 × LGALS3 in the test data set.

The training data set and test data set were divided into the two subgroups by the median scores of Z_3_ by 3.22 and Z_4_ by 0.102. The subgroups with Z_3_ ≥ 3.22 (HR = 1.549, P = 0.0253) and Z_4_ ≥ 0.102 (HR = 1.31, P = 0.0157), showed poor prognosis for OS in each data set (Fig 4A and 4B). These results suggest that the gene signature composed of common factors including GATA3 and LGALS3 would be useful to estimate prognosis of the patients with GBM. However, indices of each gene and cutoff values depended on the data set. Thus, the common formula using identical factors, indices, and cutoff values was unable to be constructed. The problem should be solved using a huge data with development of more useful and exact analytical methods in future studies.

## Discussion

Naive T cells differentiate to Th1 and Th2 cells [21,22]. Th1 cells are characterized by TBX21 and STAT4 expression [21,22]. Interferon (IFN)-γ is expressed in Th1 cells and constitutively activates type I IFN-α/β against the growth of glioma cells [23]. Further, IL-4 activates Th2 cells, which functions by eliminating extracellular parasites and producing effector cytokines [24]. The effector cells of Th2 immunity mainly comprise mast cells, IL-4/IL-5 CD4^+^ T cells, and B cells [24]. GATA3 and STAT6 play pivotal roles in Th2 cells [24]. Although the score of Th1 gene signature, including TBX21, IFNG, and IL12RB1/2, makes it difficult to estimate prognosis in GBM, higher score of Th2 gene signature, including GATA3 and IL-4, is associated with poor prognosis in GBM [15]. In the study, higher expression of GATA3, IL18R1, and TGFB3 were also associated with poor prognosis in the training data set (S4C–S4E Fig), as well as CSF2 and IL-3/4/5/6/9/13 in the test data set (S6A, S6C and S6E–S6I Fig). The Th1/Th2 lineage is developmentally different from the Th17 lineage [25]. TBX21, GATA3, and retinoic acid receptor (RAR)-related orphan receptor gamma thymus (RORC) stimulate CD4^+^ cells to differentiate into Th17 cells, defined by IL-17 production [26–28]. Th17 cells also produce IL-2, which is required for generation and maintenance of Tregs. However, IL-2 inhibits Th17 differentiation [28]. Dysregulation of Th17 cells causes malfunction of Tregs by decreasing TGF-β signaling [29,30]. In the context of Treg differentiation as described above, higher expression of TGFB1 was also associated with poor prognosis in the study (S4F Fig and S6K Fig). Furthermore, higher expression of CD163, FOXP3, and TGFB3 showed poor prognosis in the training data set (S4A, S4B and S4E Fig), but not in the test data set. Similarly, IL4 expression also showed poor prognosis in the test data set (S6F Fig).

PD-1 is significantly correlated with genes including CD40, ICOS, IDO1, SATB1 and TGFB1, and other immune checkpoint molecules including CD276, CTLA4, LAG3, and TIM3, which represents an anticancer agent [31]. The higher expression of PD-1 is associated with poor prognosis in patients with diffuse gliomas [31]. The low score of Th2 gene signature with lower expression of PD-L1, PD-L2, and PD-1 is associated with good prognosis in GBM [15]. In the study, lower expression of PD-1 and PD-1 ligands were associated with good prognosis in the training data set (Fig 1E) and the test data set (Fig 3H and 3I), respectively. In the study, lower expression of STAT1 for stimulatory checkpoint was associated with poor prognosis (S4K and S6S Figs), suggesting the suppression of stimulatory checkpoint activity as a prognosis marker candidate.

CD276 (B7-H3) is an immune checkpoint molecule that belongs to the CD28 family, which plays pivotal roles in T-cell suppression in glioma [32]. The higher expression of CD276 showed poor prognosis in glioma patients from CGGA and TCGA [33]; this was also consistent with the present data (Figs 1C, 2E and 3D). The 4IgB7H3 isoform is a candidate of therapeutic target in GBM [34]. Isocitrate dehydrogenase (IDH) mutation also seems to influence differential expression of CD276 between the grade II and higher-grade gliomas [35]. Gene ontology analysis reveals that CD276 is associated with immune response, cell cycle, cell proliferation, and Toll-like receptor signaling [35]. CD276 also discriminates endothelial cells resected from malignant tissues and normal tissues [36]. Furthermore, in addition to the advanced colorectal and breast cancers, CD276-positive circulating endothelial cells also occur in higher frequencies in patients with GBM [36]. Expression analysis represents the marked increase of GATA3 expression in phosphate-activated glutaminase-expressing GBM cell line and GBM patients [37]. In this study, higher expression of GATA3 was associated with poor prognosis in the training data set (S4C Fig), but not in the test data set (S6Q Fig). Galectin-3, a glioma-related marker encoded by LGALS3, is a β-galactosidase-binding lectin that is important in cell proliferation, adhesion, and apoptosis [38]. Galectin-3 is activated in microglia and macrophages according to the progression of glioma, however, it is not expressed in oligodendrocytic cells representing the early stage of glioma tumorigenesis [38]. In this study, lower expression of LGALS3 (galectin-3) and LGALS9 (galectin-9) were associated with a poor prognosis in the test data set (Fig 3G and 3J), but not in the training data set.

In summary, we have demonstrated that a single gene CD276 (B7-H3) and the gene signature composed of GATA3 and LGALS3 would be promising marker candidates for prognoses in GBM. Interestingly, a combination of the expression levels of GATA3 and LGALS3 enables prognosis prediction in GBM, but each gene individually is not a single marker. In addition, indices of each gene in the prediction formulas have distinct eigenvalues based on the data set, which should be further analyzed in future. However, the aim of this study, a detection for diagnosis and/or prognosis marker candidates for GBM, namely, CD276, GATA3, and LGALS3, would have been achieved successfully by using of their expression data, clinical information, and multivariable analyses. Besides, we also found the second candidate of GBM diagnosis/prognosis markers, including TIGIT, HAVCR2, PDCD1, TIGIT, and TNFRSF14. These genes were associated with patients’ survival and genetically interacted within a complex network hub, suggesting a possibility of simple diagnosis in GBM. Especially, it is of great importance that higher expression of the genes related to Th2 cells and stimulatory checkpoint molecules and lower expression of the Th1-related genes resulted in worse prognoses in the two independent GBM data sets. In addition, higher expression of the Treg-related genes also tended to show poor prognosis. These results could provide promising marker candidates for cancer immunotherapies, especially involving the inhibitory checkpoint, and would also make way for understanding and developing target therapies and pathways in GBM.

## Supporting information

S1 Fig Glioblastoma multiforme (GBM) data set used in this study. (**A-B**) Construction of the training data set and the test data set for GBM used in this study. (**A**) Training data set (N = 158). (**B**) Test data set (N = 413). Both data set are derived from The Cancer Genome Atlas (TCGA) and are independent of each other. The training data was used for initial analysis, and furthermore, the test data set was used for validations of results from the training data set. (**C-D**) Overall survival (OS) distributions of the total samples in the training data set and test data set of GBM. (**C**) Training data set. (**D**) Test data set.
(TIF)

S2 Fig Cox hazard regression analysis for 21 immunosuppressive genes in the training data set of glioblastoma multiform. (**A**) Coefficient value. (**B**) Hazard ratio.
(TIF)

S3 Fig Cox hazard regression analysis for 67 cancer immunotherapy-related genes in the training data set of glioblastoma multiform. (**A**) Coefficient values. (**B**) Hazard ratios.
(TIF)

S4 Fig Kaplan-Meier survival analysis for immunosuppressive genes in the training data set of glioblastoma multiform. Numbers in the parentheses indicate the threshold of gene expression. (**A**) CD163. (**B**) FOXP3. (**C**) GATA3. (**D**) IL18R1. (**E**) TGFB3. (**F**) TGFB1. (**G**) TNFRSF18. (**H**) TNFSF14. (**I**) TNFSF4. (**J**) HHLA2. (**K**) STAT1. (**L**) TBX21. High and low indicate subgroups with over and under the threshold. OS, overall survival. HR, hazard ratio. Subgroups were divided by the median expression of genes.
(TIF)

S5 Fig Cox hazard regression analysis for 67 cancer immunotherapy-related genes in the test data set of glioblastoma multiform. (**A**) Coefficient values. (**B**) Hazard ratios.
(TIF)

S6 Fig Kaplan-Meier survival analysis for immunosuppressive genes in the test data set of glioblastoma multiform. Numbers in the parentheses indicate the threshold of gene expression. (**A**) CSF2. (**B**) IL12RB2. (**C**) IL13. (**D**) IL2RB. (**E**) IL3. (**F**) IL4. (**G**) IL5. (**H**) IL6. (**I**) IL9. (**J**) TBX21. (**K**) TNF. (**L**) TNFRSF18. (**M**) TNFRSF4. (**N**) CD3D. (**O**) CD3E. (**P**) CD3G. (**Q**) GATA3. (**R**) LTA. (**S**) STAT1. (**T**) STAT4. (**U**) TGFB1. High and low indicate subgroups with over and under the threshold. OS, overall survival. HR, hazard ratio. Subgroups were divided by the median expression of genes.
(PDF)

S7 Fig Kaplan-Meier survival analysis for cancer immunotherapy-related genes in the training data set and test data set of glioblastoma multiform. (**A**) Kaplan-Meier survival analysis using the Z_1_ score (= -0.064) in the test data set. (**B**) Kaplan-Meier survival analysis using the Z_2_ score (= -11.181) in the training data set. OS, overall survival. HR, hazard ratio. Subgroups were divided by the median scores of Z_1_ and Z_2_.
(TIF)

S1 Table List of cancer immunotherapy-related genes. (DOCX)
