# Supplementary figures and images for: CD276 and the gene signature composed of GATA3 and LGALS3 enable prognosis prediction of glioblastoma multiforme
Source: PLoS One. 2019 May 10;14(5):e0216825. doi: 10.1371/journal.pone.0216825 (PMC6510475; doi:10.1371/journal.pone.0216825)

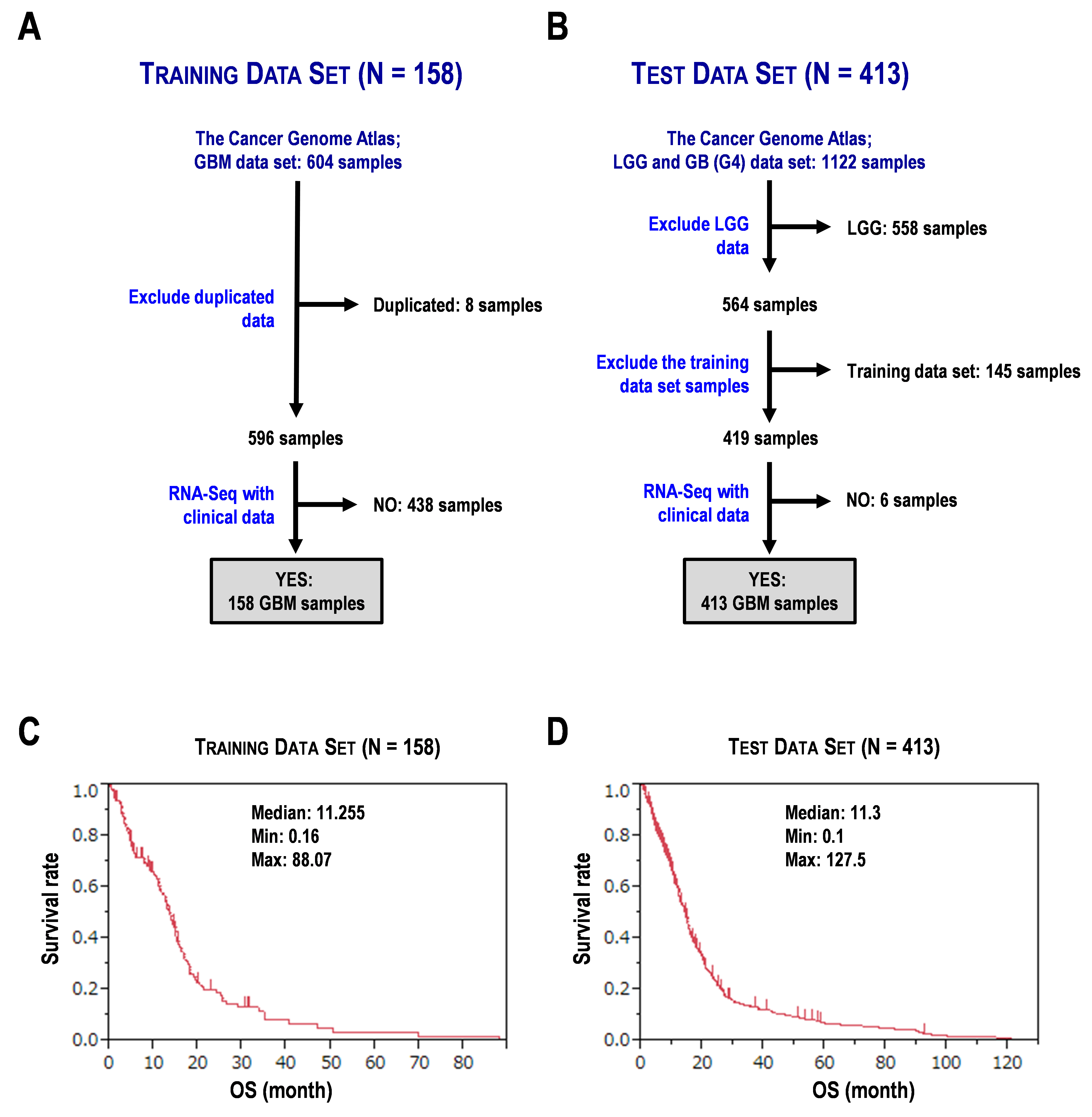

Supplement: S1 Fig — (A-B) Construction of the training data set and the test data set for GBM used in this study. (A) Training data set (N = 158). (B) Test data set (N = 413). Both data set are derived from The Cancer Genome Atlas (TCGA) and are independent of each other. The training data was used for initial analysis, and furthermore, the test data set was used for validations of results from the training data set. (C-D) Overall survival (OS) distributions of the total samples in the training data set and test data set of GBM. (C) Training data set. (D) Test data set. (TIF) [file pone.0216825.s001.tif]

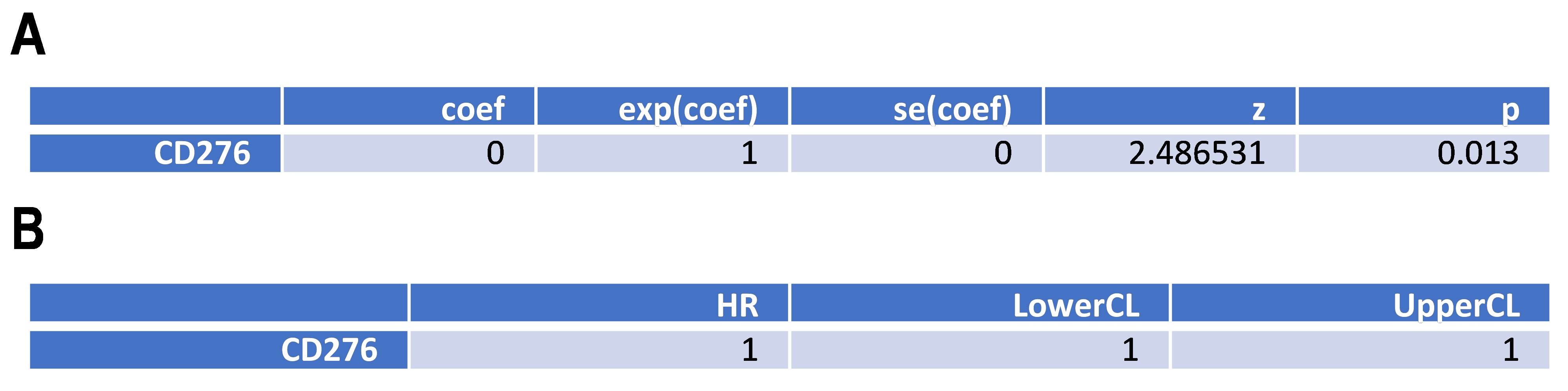

Supplement: S2 Fig — (A) Coefficient value. (B) Hazard ratio. (TIF) [file pone.0216825.s002.tif]

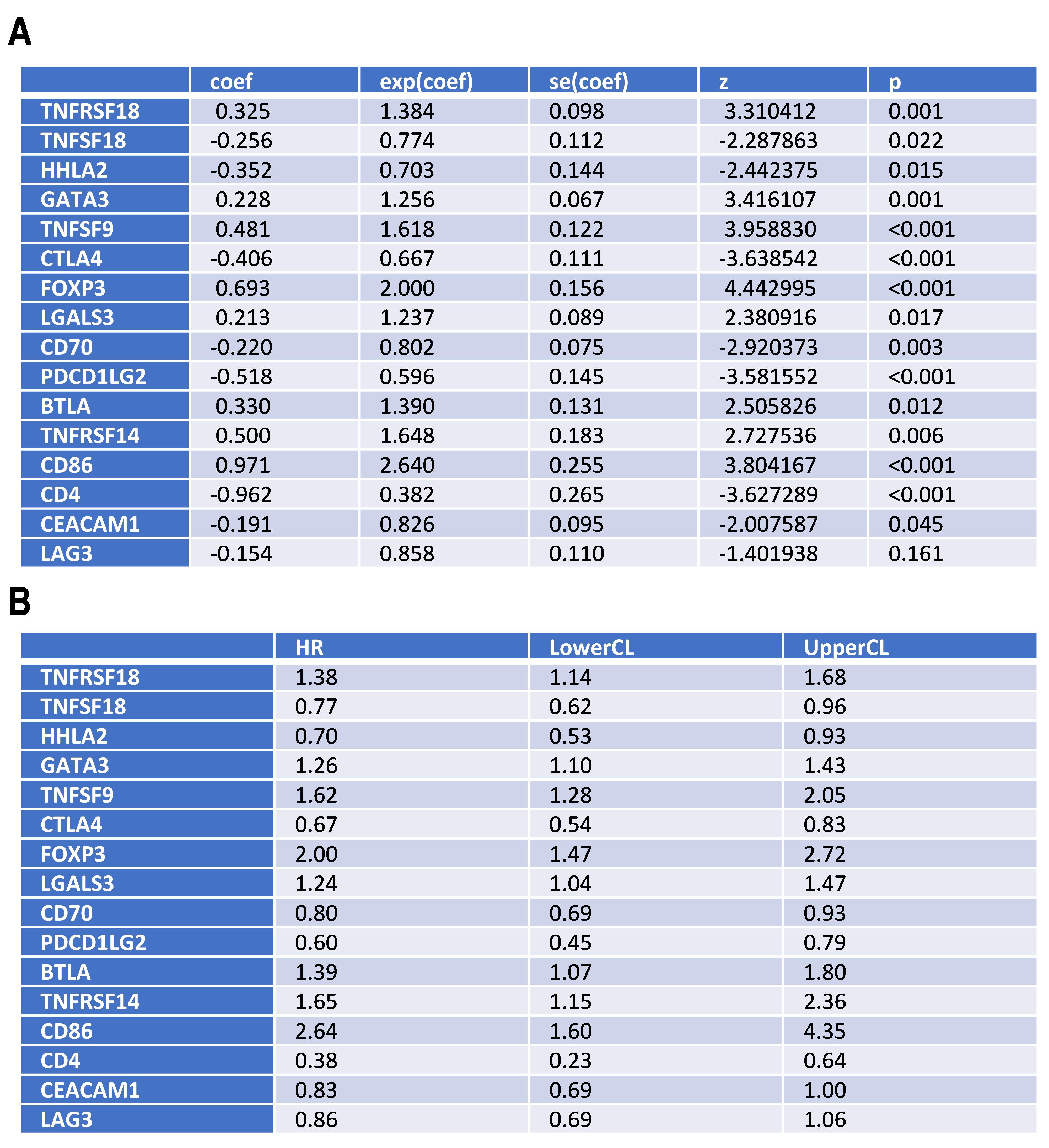

Supplement: S3 Fig — (A) Coefficient values. (B) Hazard ratios. (TIF) [file pone.0216825.s003.tif]

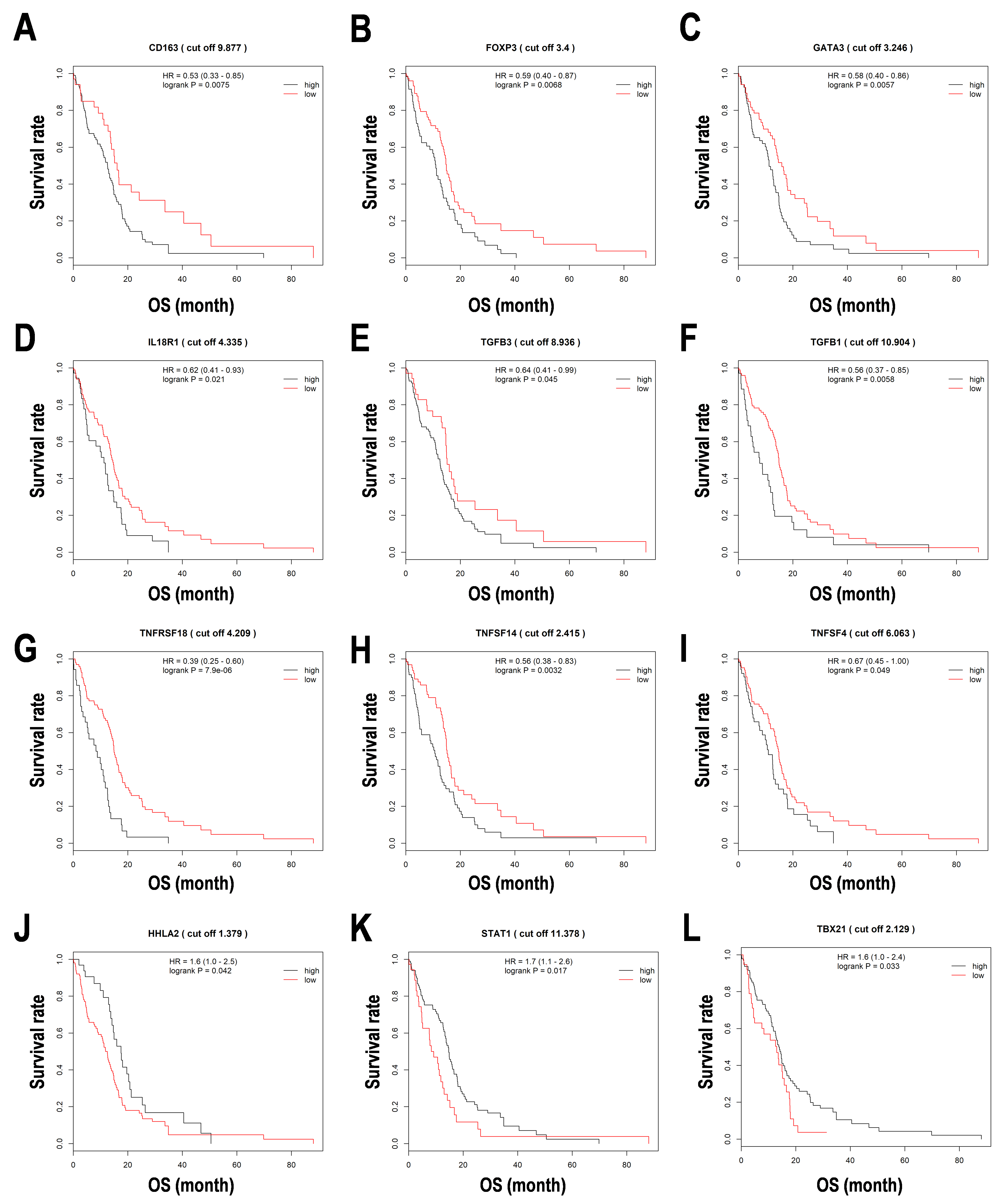

Supplement: S4 Fig — (A) CD163. (B) FOXP3. (C) GATA3. (D) IL18R1. (E) TGFB3. (F) TGFB1. (G) TNFRSF18. (H) TNFSF14. (I) TNFSF4. (J) HHLA2. (K) STAT1. (L) TBX21. High and low indicate subgroups with over and under the threshold. OS, overall survival. HR, hazard ratio. Subgroups were divided by the median expression of genes. (TIF) [file pone.0216825.s004.tif]

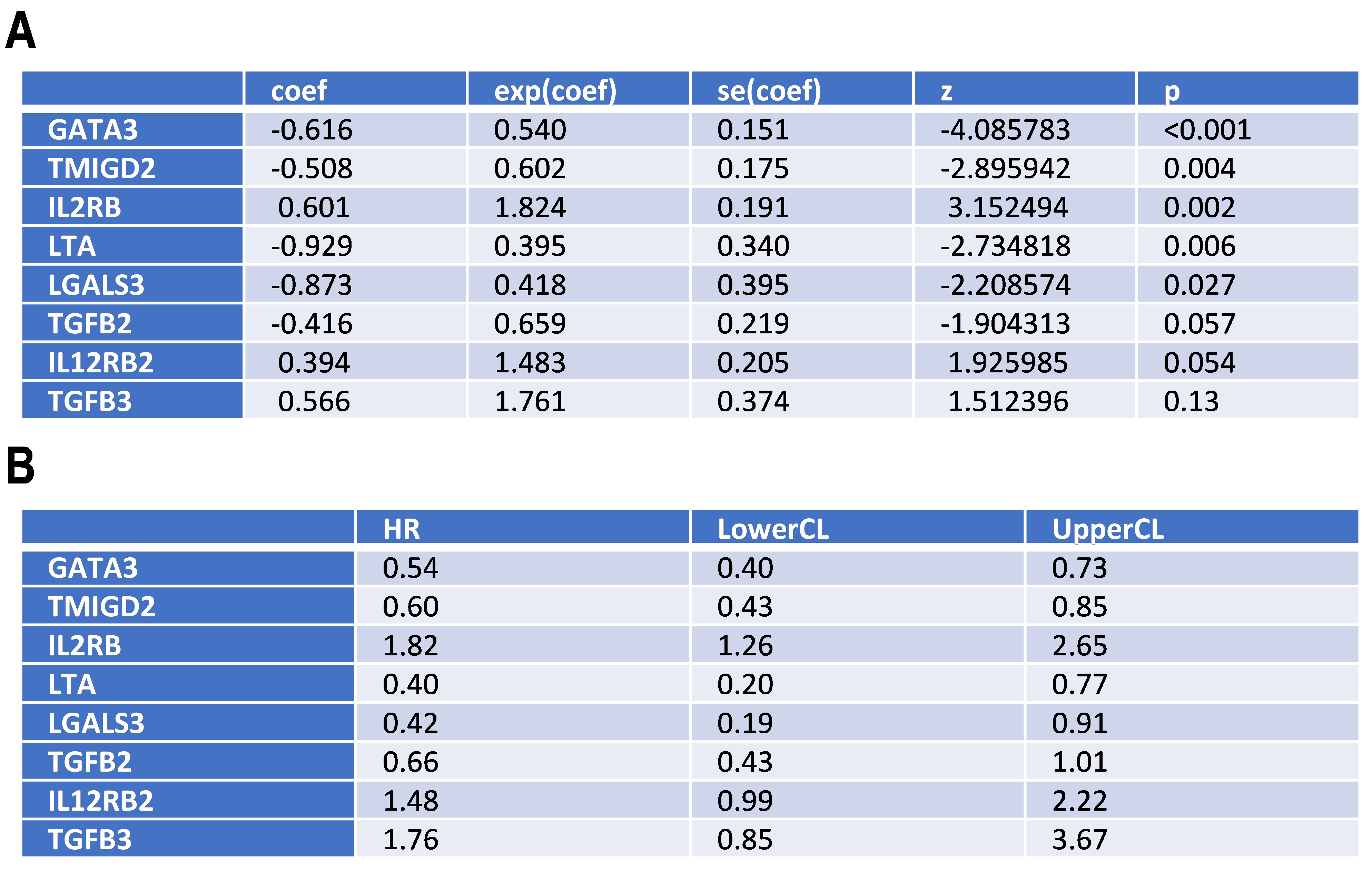

Supplement: S5 Fig — (A) Coefficient values. (B) Hazard ratios. (TIF) [file pone.0216825.s005.tif]

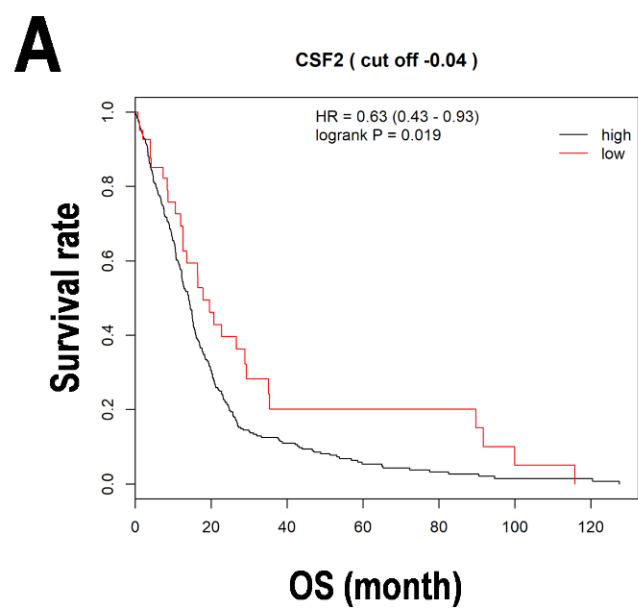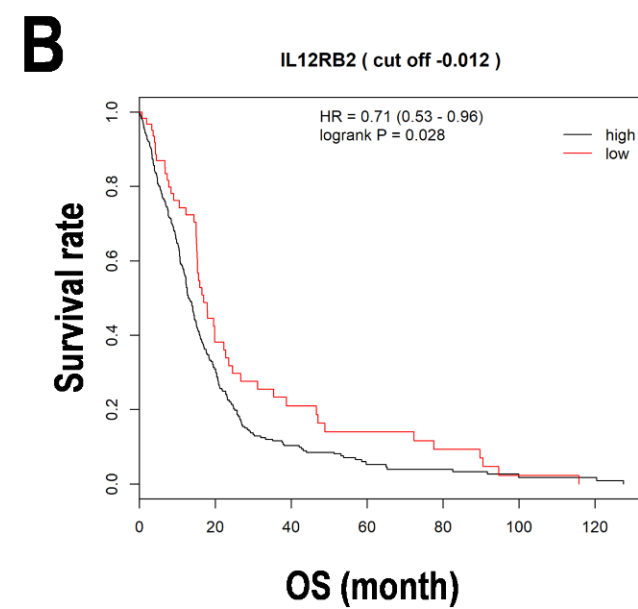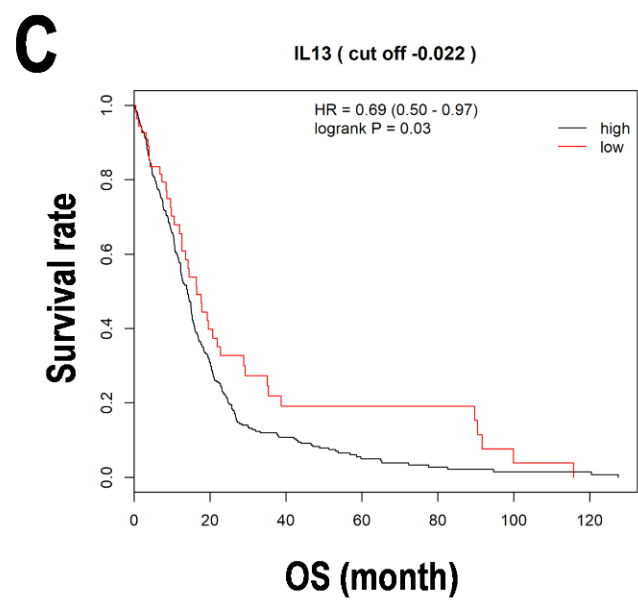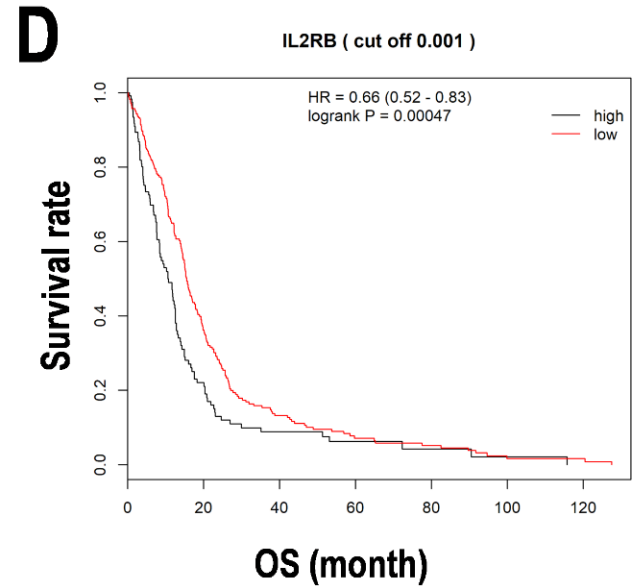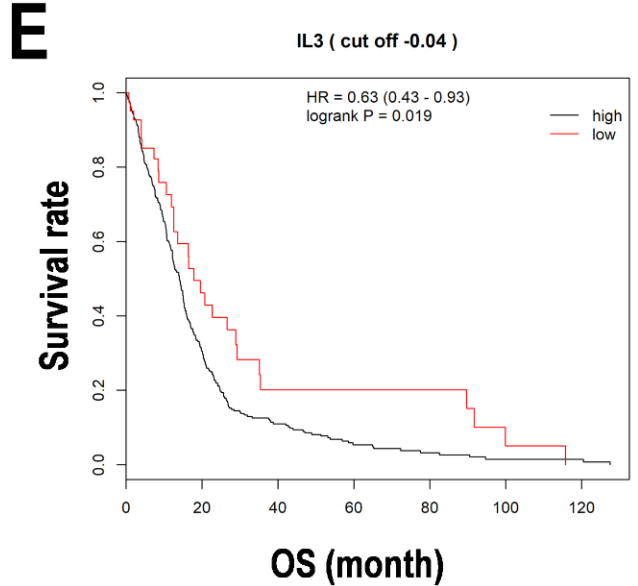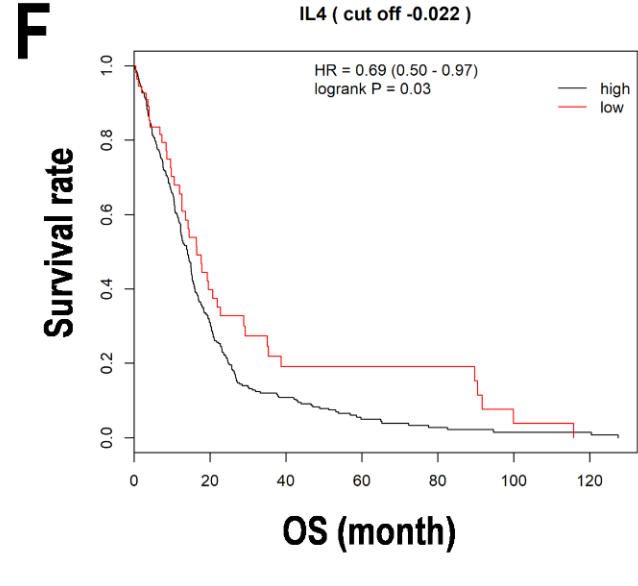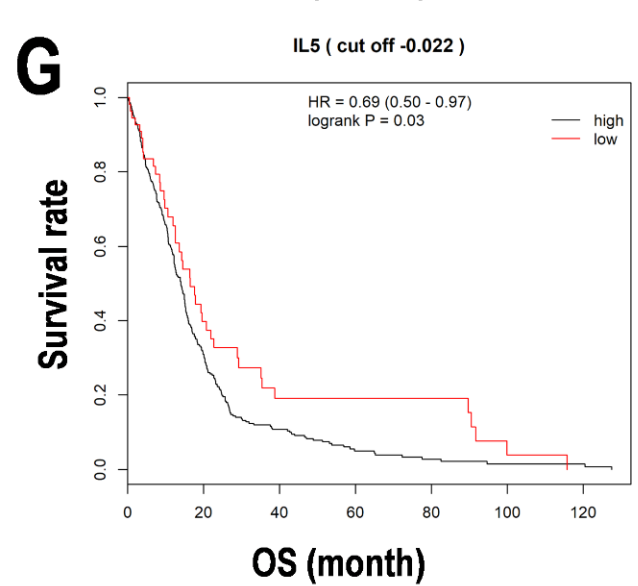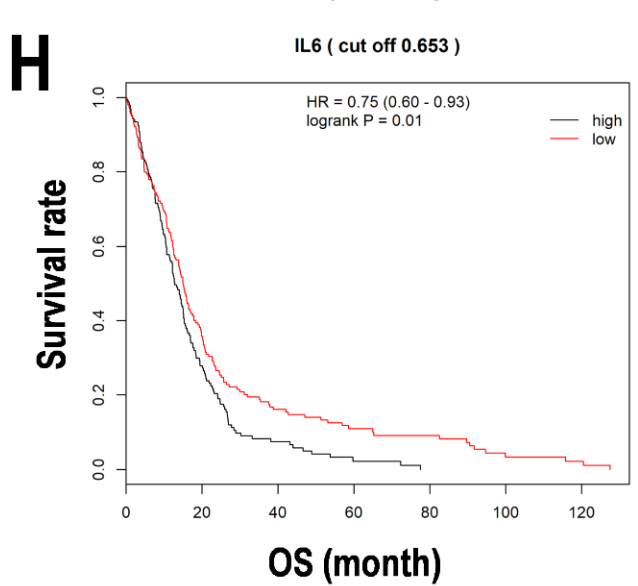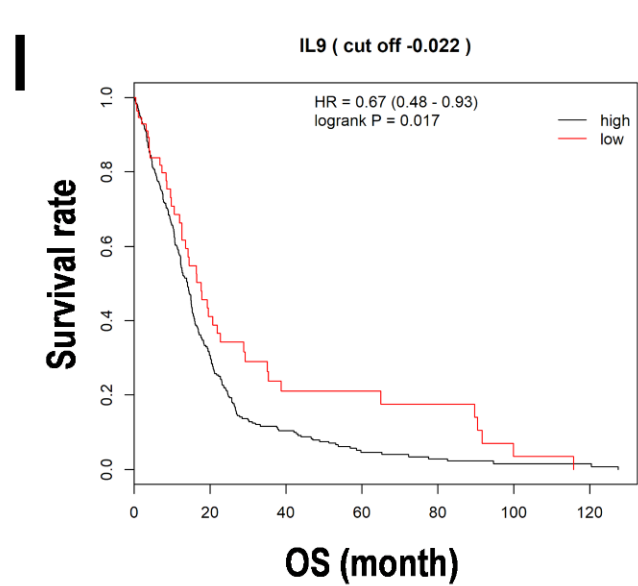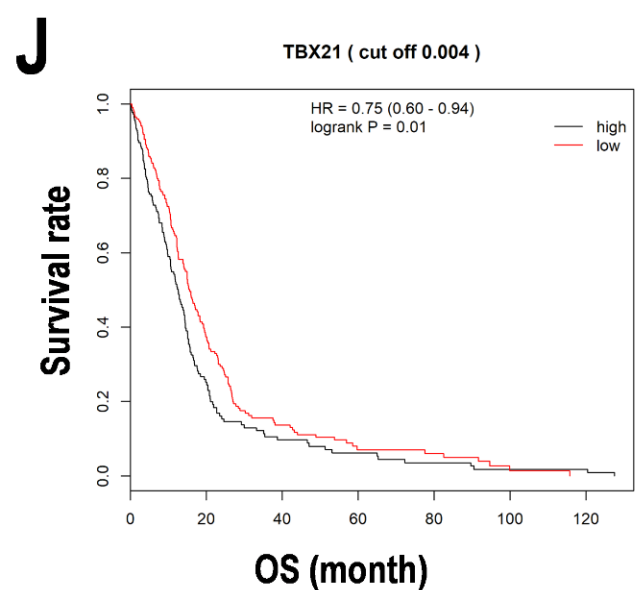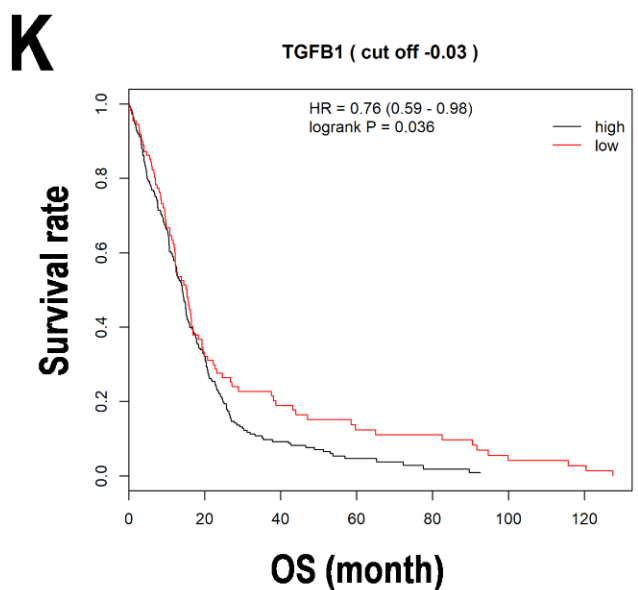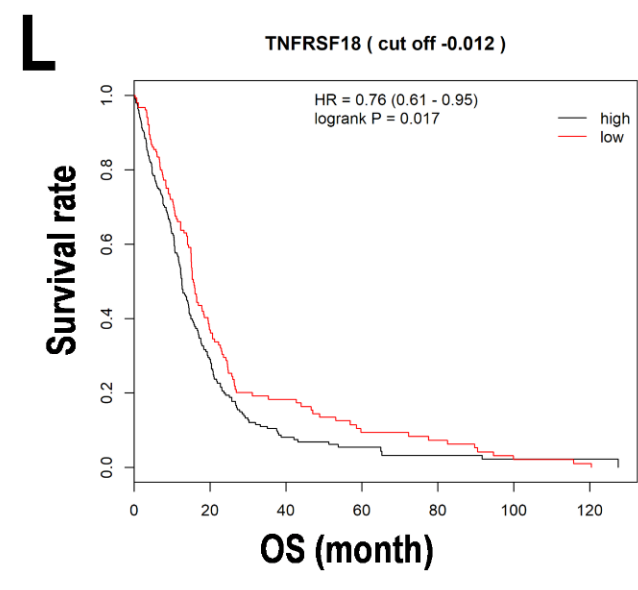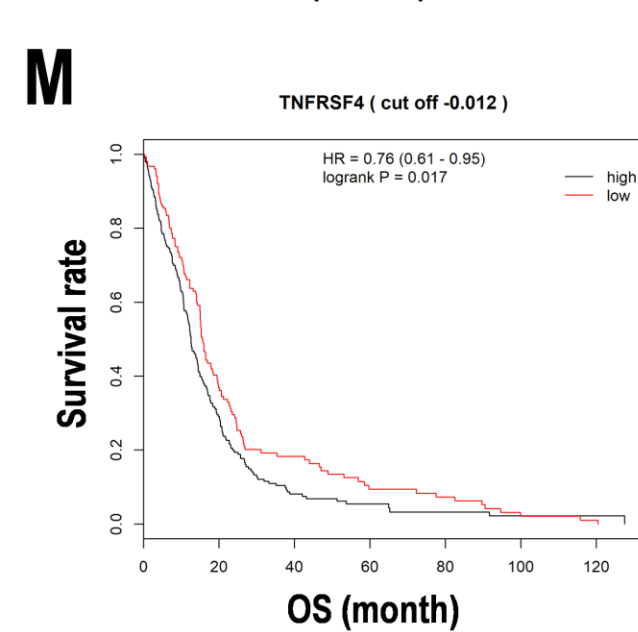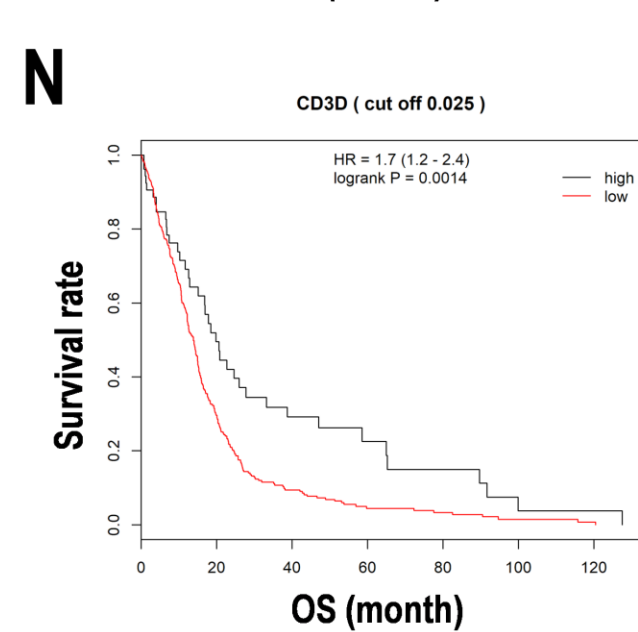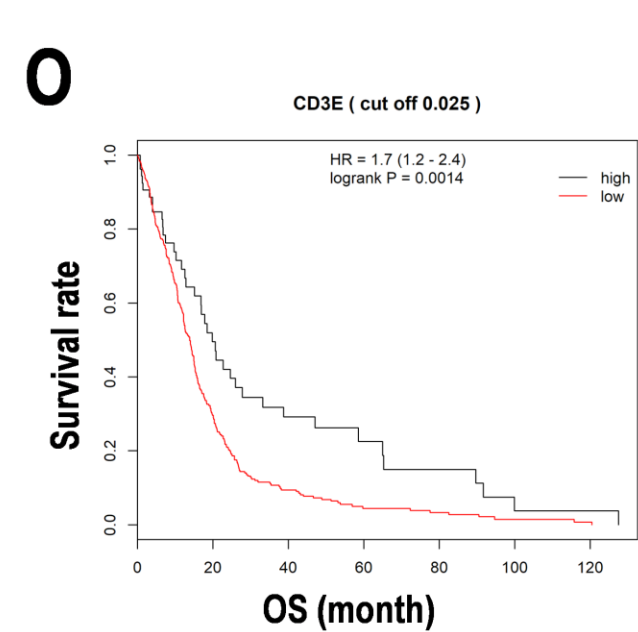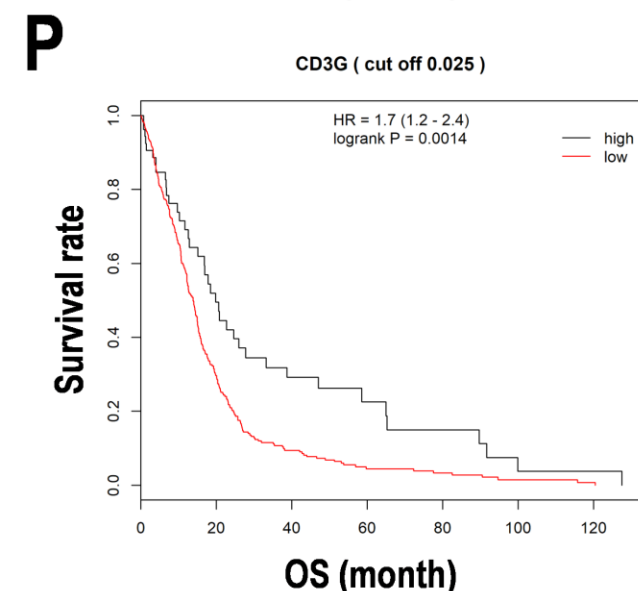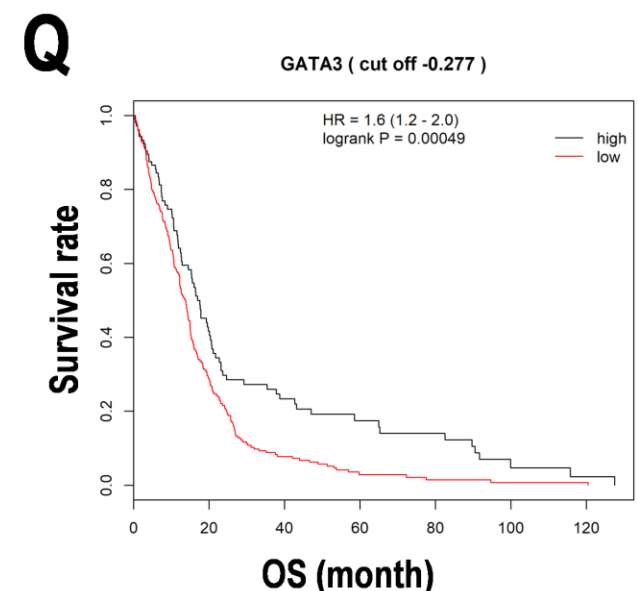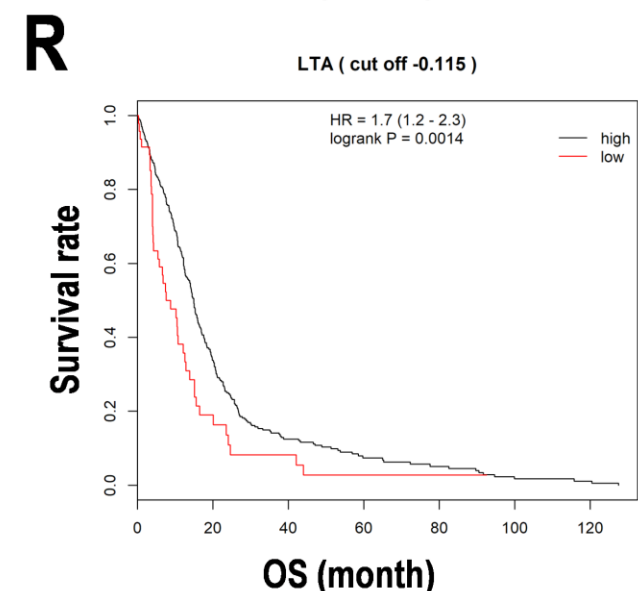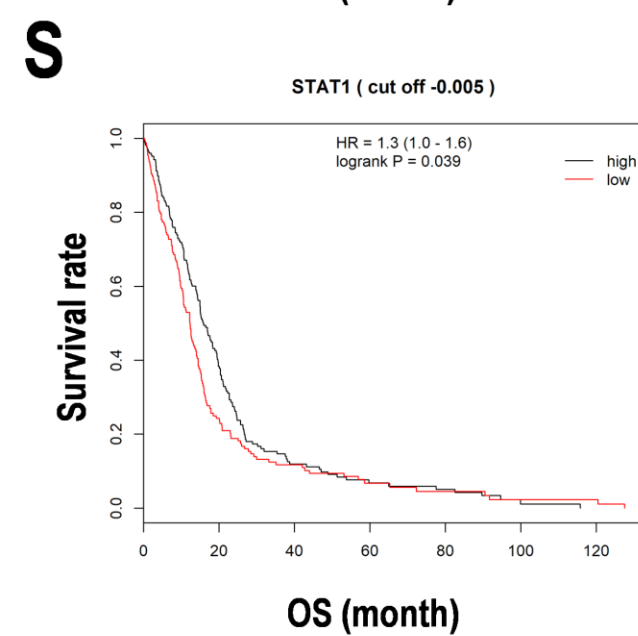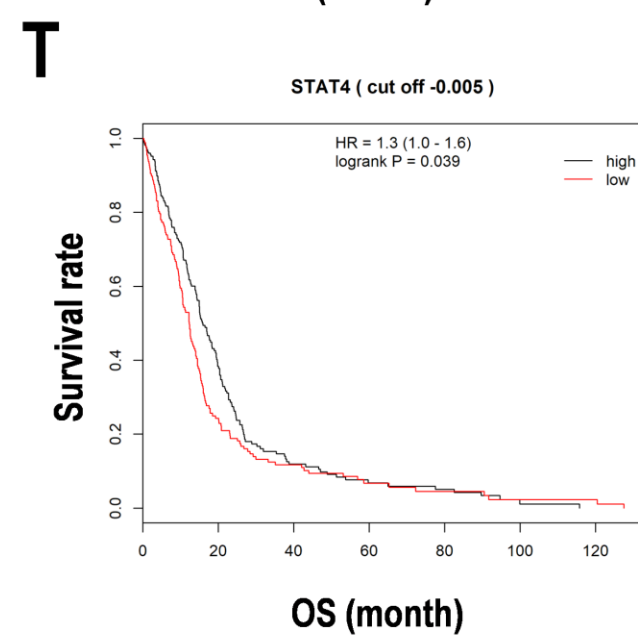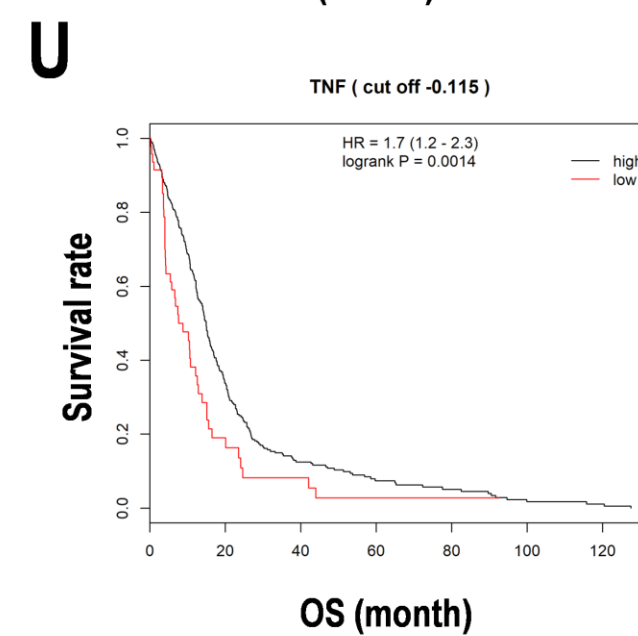

Supplement: S6 Fig — Numbers in the parentheses indicate the threshold of gene expression. (A) CSF2. (B) IL12RB2. (C) IL13. (D) IL2RB. (E) IL3. (F) IL4. (G) IL5. (H) IL6. (I) IL9. (J) TBX21. (K) TNF. (L) TNFRSF18. (M) TNFRSF4. (N) CD3D. (O) CD3E. (P) CD3G. (Q) GATA3. (R) LTA. (S) STAT1. (T) STAT4. (U) TGFB1. High and low indicate subgroups with over and under the threshold. OS, overall survival. HR, hazard ratio. Subgroups were divided by the median expression of genes. (PDF) [file pone.0216825.s006.pdf]

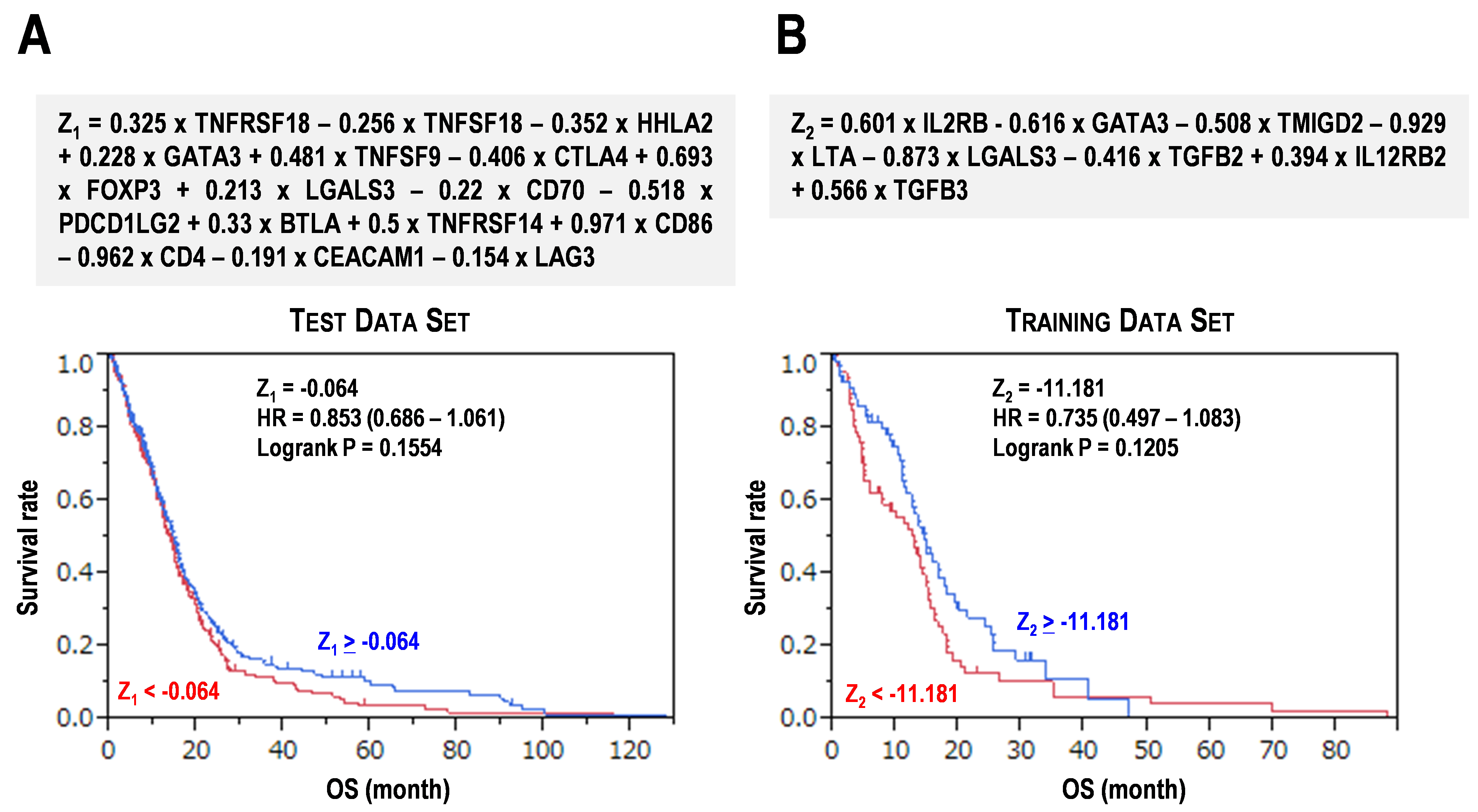

Supplement: S7 Fig — (A) Kaplan-Meier survival analysis using the Z1 score (= -0.064) in the test data set. (B) Kaplan-Meier survival analysis using the Z2 score (= -11.181) in the training data set. OS, overall survival. HR, hazard ratio. Subgroups were divided by the median scores of Z1 and Z2. (TIF) [file pone.0216825.s007.tif]
