# Supplementary material for: CD276 and the gene signature composed of GATA3 and LGALS3 enable prognosis prediction of glioblastoma multiforme
Source: PLoS One. 2019 May 10;14(5):e0216825. doi: 10.1371/journal.pone.0216825 (PMC6510475; doi:10.1371/journal.pone.0216825)
Supplement: S1 Table — (DOCX) [file pone.0216825.s008.docx]

| **S1 Table. List of cancer immunotherapy-related genes** | | | | | | | | | |
| --- | --- | --- | --- | --- | --- | --- | --- | --- | --- |
| **Accession No** | **Symbol** | **Description** | **Type 1 T helper** | **Type 2 T helper** | **Regulatory T** | **Stimulatory checkpoint** | **Inhibitory checkpoint** | **Contribution** | **References** |
| **NM_006139** | **CD28** | **CD28 molecule** | **●** | **●** |  | **●** |  | **3** | **10, 47** |
| **NM_000732** | **CD3D** | **CD3d molecule** | **●** | **●** |  |  |  | **2** | **50, 80** |
| **NM_000733** | **CD3E** | **CD3e molecule** | **●** | **●** |  |  |  | **2** | **50, 80** |
| **NM_000073** | **CD3G** | **CD3g molecule** | **●** | **●** |  |  |  | **2** | **50, 80** |
| **NM_000616** | **CD4** | **CD4 molecule** | **●** | **●** | **●** |  |  | **3** | **22, 41, 50, 53, 55, 57, 59, 68, 70, 71, 80, 93-95** |
| **NM_000074** | **CD40LG** | **CD40 ligand** | **●** | **●** |  | **●** |  | **3** | **71** |
| **NM_000758** | **CSF2** | **colony stimulating factor 2** | **●** | **●** |  |  |  | **2** | **50, 84** |
| **NM_000619** | **IFNG** | **interferon gamma** | **●** |  | **●** |  |  | **2** | **23, 51, 53, 57, 67, 68, 71, 78, 79** |
| **NM_001559** | **IL12RB2** | **interleukin 12 receptor subunit beta 2** | **●** |  |  |  |  | **1** | **39, 78** |
| **NM_003855** | **IL18R1** | **interleukin 18 receptor 1** | **●** | **●** |  |  |  | **2** | **58** |
| **NM_000586** | **IL2** | **interleukin 2** | **●** |  |  |  |  | **1** | **41, 68, 76** |
| **NM_000588** | **IL3** | **interleukin 3** | **●** | **●** |  |  |  | **2** | **70** |
| **NM_000595** | **LTA** | **lymphotoxin alpha** | **●** |  |  |  |  | **1** | **40** |
| **NM_007315** | **STAT1** | **signal transducer and activator of transcription 1** | **●** |  |  |  |  | **1** | **69** |
| **NM_003151** | **STAT4** | **signal transducer and activator of transcription 4** | **●** |  |  |  |  | **1** | **71, 83** |
| **NM_013351** | **TBX21** | **T-box 21** | **●** |  |  |  |  | **1** | **27, 71, 78, 80** |
| **NM_000594** | **TNF** | **tumor necrosis factor** | **●** |  |  |  |  | **1** | **53** |
| **NM_002051** | **GATA3** | **GATA binding protein 3** |  | **●** |  |  |  | **1** | **24, 27, 37, 70, 71, 78** |
| **NM_000572** | **IL10** | **interleukin 10** |  | **●** | **●** |  |  | **2** | **57, 93** |
| **NM_002188** | **IL13** | **interleukin 13** |  | **●** |  |  |  | **1** | **66, 70, 71** |
| **NM_000589** | **IL4** | **interleukin 4** |  | **●** | **●** |  |  | **2** | **66, 70, 76, 78, 80** |
| **NM_000879** | **IL5** | **interleukin 5** |  | **●** |  |  |  | **1** | **49, 70** |
| **NM_000600** | **IL6** | **interleukin 6** |  | **●** |  |  |  | **1** | **69, 71** |
| **NM_000590** | **IL9** | **interleukin 9** |  | **●** |  |  |  | **1** | **59, 76** |
| **NM_003153** | **STAT6** | **signal transducer and activator of transcription 6** |  | **●** |  |  |  | **1** | **66** |
| **NM_003239** | **TGFB3** | **transforming growth factor beta 3** |  | **●** | **●** |  |  | **2** | **73** |
| **NM_004244** | **CD163** | **CD163 molecule** |  |  | **●** |  |  | **1** | **88** |
| **NM_014143** | **CD274** | **CD274 molecule** |  |  | **●** |  | **●** | **2** | **5, 14, 15, 49, 54, 57, 60, 68, 72, 78, 82, 94** |
| **NM_171827** | **CD8A** | **CD8a molecule** |  |  | **●** |  |  | **1** | **89, 93** |
| **NM_005214** | **CTLA4** | **cytotoxic T-lymphocyte associated protein 4** |  |  | **●** |  | **●** | **2** | **47, 49, 54-56, 60, 72, 79, 82** |
| **NM_014009** | **FOXP3** | **forkhead box P3** |  |  | **●** |  |  | **1** | **90, 91, 93-95** |
| **NM_005018** | **PDCD1** | **programmed cell death 1** |  |  | **●** |  | **●** | **2** | **7, 15, 31, 41, 47, 49, 54, 56, 60, 72, 77-79, 81** |
| **NM_025239** | **PDCD1LG2** | **programmed cell death 1 ligand 2** |  |  | **●** |  | **●** | **2** | **78** |
| **NM_000660** | **TGFB1** | **transforming growth factor beta 1** |  |  | **●** |  |  | **1** | **92-95** |
| **NM_003238** | **TGFB2** | **transforming growth factor beta 2** |  |  | **●** |  |  | **1** | **94, 95** |
| **NM_006566** | **CD226** | **CD226 molecule** |  |  |  | **●** |  | **1** | **47, 48** |
| **NM_001242** | **CD27** | **CD27 molecule** |  |  |  | **●** |  | **1** | **68** |
| **NM_025240** | **CD276** | **CD276 molecule** |  |  |  | **●** | **●** | **2** | **32-36, 64** |
| **NM_152854** | **CD40** | **CD40 molecule** |  |  |  | **●** |  | **1** | **39, 68** |
| **NM_001252** | **CD70** | **CD70 molecule** |  |  |  | **●** |  | **1** | **42** |
| **NM_005191** | **CD80** | **CD80 molecule** |  |  |  | **●** | **●** | **2** | **39** |
| **NM_007072** | **HHLA2** | **HERV-H LTR-associating 2** |  |  |  | **●** |  | **1** | **61** |
| **NM_012092** | **ICOS** | **inducible T-cell costimulator** |  |  |  | **●** |  | **1** | **55** |
| **NM_000878** | **IL2RB** | **interleukin 2 receptor subunit beta** |  |  |  | **●** |  | **1** | **62** |
| **NM_006505** | **PVR** | **poliovirus receptor** |  |  |  | **●** | **●** | **2** | **77** |
| **NM_144615** | **TMIGD2** | **transmembrane and immunoglobulin domain containing 2** |  |  |  | **●** |  | **1** | **61** |
| **NM_003820** | **TNFRSF14** | **TNF receptor superfamily member 14** |  |  |  | **●** | **●** | **2** | **75** |
| **NM_004195** | **TNFRSF18** | **TNF receptor superfamily member 18** |  |  |  | **●** |  | **1** | **55, 63, 80** |
| **NM_003327** | **TNFRSF4** | **TNF receptor superfamily member 4** |  |  |  | **●** |  | **1** | **80** |
| **NM_001561** | **TNFRSF9** | **TNF receptor superfamily member 9** |  |  |  | **●** |  | **1** | **80** |
| **NM_003807** | **TNFSF14** | **tumor necrosis factor superfamily member 14** |  |  |  | **●** |  | **1** | **46** |
| **NM_005092** | **TNFSF18** | **tumor necrosis factor superfamily member 18** |  |  |  | **●** |  | **1** | **63** |
| **NM_003326** | **TNFSF4** | **tumor necrosis factor superfamily member 4** |  |  |  | **●** |  | **1** | **42, 55** |
| **NM_003811** | **TNFSF9** | **tumor necrosis factor superfamily member 9** |  |  |  | **●** |  | **1** | **64** |
| **NM_181780** | **BTLA** | **B and T lymphocyte associated** |  |  |  |  | **●** | **1** | **45** |
| **NM_007053** | **CD160** | **CD160 molecule** |  |  |  |  | **●** | **1** | **45** |
| **NM_175862** | **CD86** | **CD86 molecule** |  |  |  |  | **●** | **1** | **39** |
| **NM_198196** | **CD96** | **CD96 molecule** |  |  |  |  | **●** | **1** | **47, 56** |
| **NM_001712** | **CEACAM1** | **carcinoembryonic antigen related cell adhesion molecule 1** |  |  |  |  | **●** | **1** | **44, 60** |
| **NM_032782** | **HAVCR2** | **hepatitis A virus cellular receptor 2** |  |  |  |  | **●** | **1** | **60, 72** |
| **NM_002164** | **IDO1** | **indoleamine 2,3-dioxygenase 1** |  |  |  |  | **●** | **1** | **12, 68** |
| **NM_002286** | **LAG3** | **lymphocyte activating 3** |  |  |  |  | **●** | **1** | **62** |
| **NM_002306** | **LGALS3** | **galectin 3** |  |  |  |  | **●** | **1** | **38, 43, 52** |
| **NM_002308** | **LGALS9** | **galectin 9** |  |  |  |  | **●** | **1** | **43, 51** |
| **NM_173799** | **TIGIT** | **T-cell immunoreceptor with Ig and ITIM domains** |  |  |  |  | **●** | **1** | **47, 48, 56, 72, 77** |
| **NM_022153** | **VISTA** | **V-set immunoregulatory receptor** |  |  |  |  | **●** | **1** | **81, 94** |
| **NM_024626** | **VTCN1** | **V-set domain containing T cell activation inhibitor 1** |  |  |  |  | **●** | **1** | **65** |
| **NOTE: The references 39-95 were listed in the Supplementary References.** | | | | | | | | | |
